# Development and Clinical Efficacy of the Application for Articulation Therapy‐Thai (AAT‐T) for Thai Children With Cleft Lip and Palate

**DOI:** 10.1002/cre2.70194

**Published:** 2025-07-31

**Authors:** Benjamas Prathanee, Sumita Duangprasert, Sasalaksamon Chanachai, Patorn Piromchai

**Affiliations:** ^1^ Department of Otorhinolaryngology, Faculty of Medicine Khon Kaen University Thailand; ^2^ Mekong Health Science Research Institute Khon Kaen University Thailand

**Keywords:** application, articulation therapy, cleft lip and palate, telepractice

## Abstract

**Objectives:**

Mobile applications are becoming essential for speech therapy, especially in areas with limited access to professional speech therapists. This technological intervention is especially pertinent during pandemics, which further restrict access to traditional therapeutic modalities. The objective of this study was to develop and evaluate a mobile application for articulation therapy, specifically designed for pediatric patients with articulation disorders associated with cleft palate.

**Material and Methods:**

The articulation exercises incorporated in the application encompassed 27 Thai initial and final consonant sounds, presented through video demonstrations and tabular reading materials. These materials included nonsense syllables, words, phrases, and sentences. The content validity was assessed using the Item‐Objective Congruence (IOC) index. Face validity and functional satisfaction were evaluated by a panel comprising four speech‐language pathologists (SLPs), five children with cleft palate with or without cleft lip (CP ± L) and their respective caregivers, and an expert in application design. The clinical efficacy of the application for speech correction was subsequently assessed in a cohort of 19 children diagnosed with CP ± L.

**Results:**

The Application for Articulation Therapy‐Thai (AAT‐T) was finalized after four iterative revisions. The IOC index for the application ranged from 0.80 to 1.00, indicating strong content validity. Functional satisfaction scores ranged from 76% to 100%, suggesting high user acceptability. Nineteen children participated in the clinical efficacy assessment; however, one child was withdrawn due to the inability to complete 6‐month follow‐up. The results demonstrated that AAT‐T significantly reduced articulation errors in connected speech (mean difference = 2.33, SD = 2.40, 95% CI = 1.14–3.53, *p* < 0.001).

**Conclusions:**

The AAT‐T application emerged as an accessible, engaging, and motivational tool for articulation practice. Its efficacy in speech correction was demonstrated.

## 1 Introduction

The prevalence of cleft lip and palate based on the meta‐analysis of the studies reviewed in each 1000 live births was 0.45 (95% CI: 0.38−0.52) (Salari et al. 2022). Speech defects in children with cleft palate with or without cleft lip (CP ± L) are frequently exhibited, particularly compensatory articulation disorders (CAD), hypernasality, voice disorders, turbulence, audible nasal emission, less understandability, and deviation acceptability (de Araújo et al. 2022; Hardin‐Jones and Jones 2005; Rullo et al. 2009; Smarius et al. 2021).

Long‐term speech defects can lead to a range of significant issues, including illiteracy (Chokbundit and Pratahnee 2016; Ingkapa and Prathanee 2016), behavior problems, depression, and anxiety (Hunt et al. 2006). Therefore, early diagnosis of compensatory articulation disorders (CAD) and the implementation of effective interventions are essential to mitigate the risk of developmental and socio‐emotional difficulties.

Several speech approaches and models have been proposed for the correction of speech disorders in children with cleft palate with or without cleft lip (CP ± L) in developed countries. However, the inaccessibility of speech services remains a critical concern in several developing nations, including Thailand (Makarabhirom et al. 2015; Prathanee et al. 2014), Myanmar, Lao People's Democratic Republic (Prathanee et al. 2020), and Mexico (Pamplona et al. 2005).

This inaccessibility is often due to various limitations, including the absence of available speech services. Furthermore, the COVID‐19 pandemic has significantly exacerbated the challenges associated with accessing speech therapy in these regions. Consequently, addressing these issues requires urgent and sustained attention to develop effective strategies for improving access to speech services.

There is a need for a software program designed for use on computers, tablets, or mobile devices specifically tailored to address this purpose. A software application focused on speech therapy for children with cleft palate with or without cleft lip (CP ± L) represents a promising strategy for facilitating speech correction.

The use of applications that incorporate sounds and animations offers distinct advantages over traditional printed materials. Most children today are more likely to engage with software programs that are user‐friendly, visually appealing, enjoyable, motivating, and exciting while they participate in activities (Dural and Ünal–Logacev 2018). These applications enable children to record and listen to sounds, providing immediate auditory feedback that facilitates the recognition and correction of phonological errors (Wafi 2003).

To the best of our knowledge, there is currently no standard articulation therapy application specifically designed for the Thai population. The objective of this study was to develop an articulation therapy application tailored for children with articulation disorders.

## 2 Methods

The development of the Application for Articulation Therapy‐Thai (AAT‐T) and its validation tests were conducted. AAT‐T was specifically designed as an application to support the therapy of children with cleft palate with or without cleft lip (CP ± L) who experience articulation disorders. The investigator collaborated with Digix Technology Company to create a flow chart for AAT‐T during interactive Zoom meetings. The company had no role in the conceptualization, design, methodology, investigation, data analysis, interpretation, or reporting of the findings in this study. All aspects of the study were conducted independently by the research team.

Feedback and insights were gathered from five experts, including four speech and language pathologists (SLPs) with over ten years of experience in speech therapy for children with CP ± L, as well as an expert in application design, to ensure the application's functionality was appropriately structured.

The content for the AAT‐T was developed to include all Thai phonemes, comprising 27 sounds (21 initial sounds and 6 final sounds). AAT‐T features eight levels of articulation exercises:

1. **Isolated Sounds**: 20 exercises focusing on individual sounds, accompanied by videos demonstrating the motion of the speech organs for each target sound.
2. **Nonsense Syllables (1 syllable)**: 20 exercises involving single nonsense syllables.
3. **Nonsense Syllables (2 syllables)**: 20 exercises featuring two nonsense syllables that share the same vowel.
4. **Nonsense Syllables (3 syllables)**: 20 exercises consisting of three nonsense syllables with the same vowel.
5. **Nonsense Syllables (2–3 syllables)**: 20 exercises that include two to three nonsense syllables with different vowels in each syllable.
6. **Word Level**: 35 exercises featuring words, which include 15 pictures and a reading list of 20 words.
7. **Short Phrases/Sentences**: 35 exercises consisting of short phrases or sentences, accompanied by 15 pictures and a reading list of 20 short phrases or sentences containing 3 to 5 syllables.
8. **Long Phrases/Sentences**: 35 exercises involving longer phrases or sentences, featuring 15 pictures and a reading list of 20 phrases or sentences containing 4 to 8 syllables.

This structured approach aims to progressively enhance articulation skills in children with CP ± L.

Children can imitate the videos or animations of speech organs provided in Levels 1–8. If a child is unable to name a picture or read the speech sample displayed on the mobile phone screen, they can still practice sound production by pressing the speaker symbol (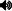). AAT‐T also includes a feature for children with literacy skills, allowing them to practice by reading the exercise worksheets. Articulation can be manually scored and recorded as correct or incorrect within the AAT‐T program, which subsequently generates a summary table of final performance upon completion of the exercises.

**Figure 1:**
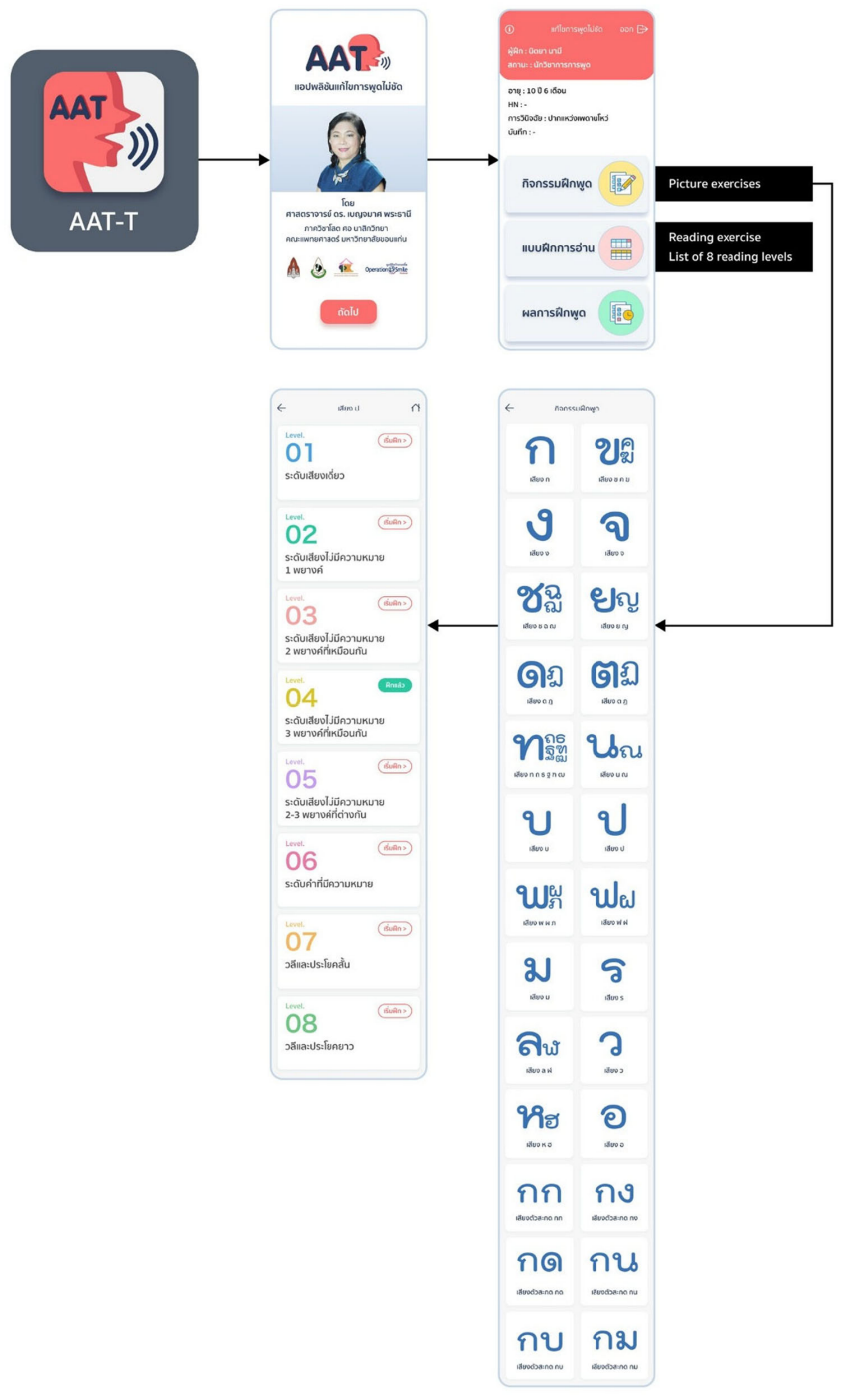
Flow chart of user interface.

The flow chart of the user interface is illustrated in Figure 1. The interface begins with the Logo Screen, which displays the app name “AAT‐T” along with a speech icon, followed by the Welcome Screen showing the project lead and institutional logos. The Main Menu includes two components: picture exercises and reading exercises. Both exercise types are organized into levels 1 through 8. Lastly, the Options Screen displays a Thai alphabet grid.

## 3 The Index of Item‐Objective Congruence

The AAT‐T was submitted to the same five experts for evaluation of the Item Objective Congruence (IOC) Index, along with their comments and suggestions. The IOC was quantified using a scoring system where −1 indicated “not appropriate,” 0 represented “not available/not sure,” and 1 signified “appropriate.” The evaluation focused on two primary areas: (1) the content and order of AAT‐T, assessing the accuracy of contexts and images, consistency of content and objectives, logical sequencing, and the appropriateness and quantity of content items; and (2) the quality of pictures, sounds, and language, evaluating the clarity of images, sound levels, image size, and font size and style. Following this assessment, AAT‐T was revised and resubmitted to the experts for rescoring, particularly for items with an IOC score of less than 0.80. The development and rationale for each version are presented in Table 1.

**Table 1:** Development and explanation of each AAT‐T version.

Interactive‐zoom meetings	Topic	Reason	Revision part
Mock presentation I (5 experts)	Flow chart or UI	UI was not clear, and some parts did not relate to the stakeholders' needs	Flow chart or UI
Mock presentation II (5 experts)	UI Revision of AAT‐T	Minimal UI Revision for more benefit of utilization and appropriation	Flow chart or UI
Mock presentation III–V (5 experts)	Utility of the 1st version	Clarified Pictures, Videos, and icons and revised to be the 2nd version	Utilization process of the 1st version
**Assessment of AAT‐T**			
Scoring index of item‐objective congruence (IOC) I (5 experts)	Utility of the 2nd version of AAT‐T	Appropriate content, order, picture, sound, and language of AAT‐T and revised to be the 3rd version	Process of utilization of AAT‐T
Scoring index of item‐objective congruence (IOC) II (5 experts)	Utility of the 3rd version of AAT‐T	Appropriate content, order, picture, sound, and language of AAT‐T and revised to be the 4th version	Process of utilization of AAT‐T
Face validity 4 SLPs, 5 children with CP ± L and their caregivers	Utility of the 4th version of AAT‐T	Clinical utility of the 4th version of AAT‐T and minimal modification to be the final version	Process of utilization of AAT‐T

The final version of the AAT‐T (4th version) was used to evaluate face validity, clinical efficacy, percentage of agreement, and caregiver satisfaction.

### 3.1 Face Validity

The face validity of AAT‐T Version IV was assessed by an additional four speech‐language pathologists (SLPs) who had worked with children with cleft palate with or without cleft lip (CP ± L) for more than 6 years, as well as five children with CP ± L (aged 5 years and 5 months to 11 years and 7 months) and five caregivers. The SLPs quantified their scores and provided suggestions, comments, and evaluations of questionnaires assessing the satisfaction of functional requirements, application functions, and usability tests. Children with CP ± L and their caregivers quantified the need, accuracy, and utility of the application functions. The suggestions and comments received were incorporated to modify and refine AAT‐T Version IV, resulting in the final version of the application.

### 3.2 Clinical Efficacy of AAT‐T for Reduction of Articulation Errors

The final version of the AAT‐T was implemented in a pre‐ and post‐prospective study to evaluate its effectiveness in speech therapy for children with cleft palate with or without cleft lip (CP ± L). The inclusion criteria were children with CP ± L who had undergone surgical repair, while the exclusion criteria were children with CP ± L who had concomitant congenital defects, global developmental delays (e.g., intellectual disability, autism, cerebral palsy), or fewer than two articulation errors (excluding/r/errors, which are the most common errors in Thai and do not have a definitive age of acquisition), or have hearing/vision impairment.

Nineteen children with cleft palate with or without cleft lip (CP ± L) who had undergone surgical repair at least 1 year prior, along with their caregivers, were enrolled in this study. One boy (A06) withdrew from the project due to the caregiver's inability to participate in the ongoing follow‐up.

**Figure 2:**
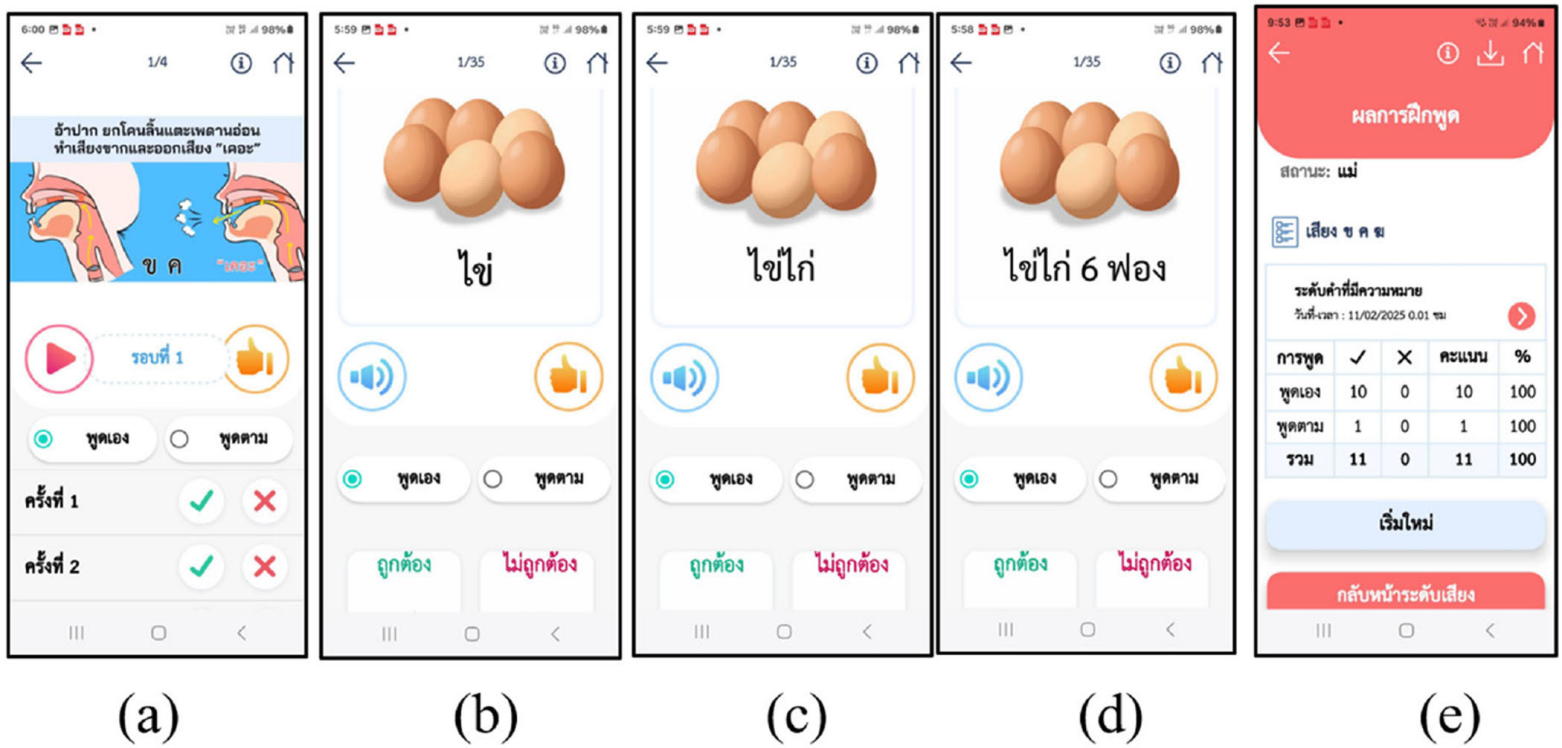
AAT‐T screen for practicing/kh/in (a) isolated sound, (b) word (egg), (c) 2‐syllable word or short phrase (chicken egg), (d) long phrase or sentence (six chicken eggs), and (e) report of score.

Eighteen children with cleft palate with or without cleft lip (CP ± L), aged 5–13 years (male‐to‐female ratio = 11:7), underwent pre‐ and post‐intervention assessments of speech parameters, including articulation, resonance, nasal emission, and turbulence using the Articulation Screening Test (Prathanee 2014). The participants received 20 sessions of speech therapy over 5 months, consisting of 15 telepractice (TP) sessions and 5 face‐to‐face sessions, utilizing the AAT‐T application via Zoom or Line applications. Figure 2 depicts the AAT‐T screen used for speech correction. A paired *t*‐test was employed to determine the statistical significance of the mean differences in pre‐ and post‐intervention articulation errors.

Each child and caregiver practiced the same sounds across all levels, including isolated sounds, nonsense syllables, words, short phrases, and long phrases/sentences. Subsequently, specific exercises targeting these sounds were assigned to caregivers for the purpose of facilitating speech correction using the AAT‐T application.

### 3.3 Percentage of Agreement

To assess the level of agreement between speech‐language pathologists (SLPs) and caregivers in scoring articulation accuracy, percentages of agreement were calculated during two face‐to‐face speech therapy sessions: (1) the first session, conducted in the second month of the project, and (2) the second session, conducted in the sixth month of the project. It is important to note that the majority of caregivers remained the same individuals between the first and second follow‐up assessments.

### 3.4 Caregivers' Satisfaction

Caregivers were asked to complete a questionnaire to assess their level of satisfaction with the speech therapy provided using the AAT‐T application at the end of the study. The questionnaire utilized a 5‐point Likert scale, with 1 representing “very little satisfaction,” 2 indicating “a little satisfaction,” 3 denoting “moderate satisfaction,” 4 signifying “much satisfaction,” and 5 corresponding to “very much satisfaction.”

### 3.5 Ethical Approval

In accordance with the Helsinki Declaration, this study was approved by the Ethics Committee for Human Research at Khon Kaen University (Approval ID: HE 644014). Written informed consent was obtained from all participants and their caregivers.

## 4 Results

### 4.1 Item Objective Congruence Index

The IOC scores for each sound across all levels, including isolated sounds, nonsense syllables, words, and phrase‐sentence levels, as well as the lists for each level in various contexts, the mean score ranged from 0.80 to 1.00.

### 4.2 Face Validity

The satisfaction scores from speech specialists regarding functional requirements ranged from 80% to 100%. The score for the functional test was a perfect 100%. Usability scores varied between 72% and 96%. Additionally, satisfaction scores from caregivers and children with cleft palate with or without cleft lip (CP ± L) were notably high, ranging from 92% to 100% for displayed functionality, achieving 100% for the functional test, and ranging from 92% to 100% for usability.

### 4.3 Agreement Between Specialists and Caregivers

The percentage of agreement between speech‐language pathologists (SLPs) and caregivers increased over time between 2 months and 6 months follow‐up, reaching 81.48%. However, there were slight declines in 13.89% of cases, significant decreases in 3.70% of cases, and no change observed in 0.93% of cases (see Table 2).

**Table 2:** Percentage of agreement between the first and second assessments.

Child code	Sounds	Percent of agreement							
		Nonsense syllable		Word		Short phrase/sentence		Long‐phrase/sentence	
		Pre	Post	Pre	Post	Pre	Post	Pre	Post
1	/k/	80	N/A	73.33	60.00	60.00	60.00	46.67	60.00
	/‐m/	100	100	100	93.33	100	100	100	100
2	/n/	100	100	100	93.33	100	93.33	100	100
	/ʔ/	100	100	100	100	93.33	100	80	100
3	/‐k/	100	93.33	93.33	93.33	73.33	93.33	80	86.67
4	/l/	93.33	100	73.33	93.33	57.14	100	80.0	73.33
5	/ŋ/	80.00	100	86.67	93.33	66.67	86.67	86.67	93.33
	/‐n/	60.00	0	N/A	26.67	40.00	13.33	35.71	53.33
6	/‐p/	66.67	N/A	93.33	N/A	66.67	N/A	33.33	N/A
7	/t_ɕ_/	93.33	100	93.33	100	93.33	100	86.67	100
	/r/	86.67	100	53.33	100	66.67	100	33.33	100
8	/d/	80.00	60.00	66.67	86.67	80.00	86.67	80.00	86.67
	/w/	20.00	86.67	40.00	66.67	53.33	73.33	53.33	93.33
9	/m/	100	100	80.00	93.33	33.33	100	13.33	93.33
	/k^h^/	46.67	93.33	73.33	86.67	80.00	93.33	40.00	93.33
10	/t_ɕ_^h^/	13.33	40.00	86.67	60.00	46.67	53.33	40.00	40.00
11	/‐t/	33.33	40.00	6.67	46.67	20.00	46.67	33.33	53.33
12	/b/	80.00	100	93.33	100	100	100	93.33	100
13	/p^h^/	100	93.33	100	100	100	93.33	100	80.00
14	/t^h^/	100	100	100	100	100	93.33	100	86.67
	/j/	100	100	100	100	100	100	100	100
15	/f/	100	100	93.33	93.33	93.33	93.33	80.00	93.33
16	/s/	100	100	93.33	100	93.33	100	100	100
17	/p/	53.33	40.00	66.67	86.67	60.00	73.33	46.67	80.00
18	/t/	100	53.33	93.33	73.33	86.67	93.33	93.33	86.67
19	/h/	86.67	86.67	53.33	100	73.33	86.67	66.67	86.67

### 4.4 Clinical Efficacy to Reduce Articulation Errors

Nineteen children participated in the clinical efficacy assessment; however, one child was withdrawn due to the inability to complete the 6‐month follow‐up. The number of articulation errors significantly decreased in the connected speech level following 15 weekly telepractice sessions and 5 monthly face‐to‐face speech therapy sessions using AAT‐T, with a mean difference of 2.33 (SD = 2.40, 95% CI = 1.14 – 3.53, *p* < 0.001) (see Table 3).

**Table 3:** Comparison of the number of articulation errors.

Pre‐Test				Posttest				Paired *t*‐test			
Min	Max	Mean	SD	Min	Max	Mean	SD	Mean difference	SD	95% CI	
										Lower	Upper
4	13	7.39	2.59	2	9	5.06	2.56	2.33	2.40	1.14	3.53

### 4.5 Caregivers' Satisfaction

The satisfaction scores at 6 months follow‐up exceeded four out of five for all items (see Table 4). In addition to the quantitative questionnaires assessing the ease of use and attractiveness of the application, individual interviews revealed that all caregivers or parents (100%) expressed satisfaction with AAT‐T. They cited benefits such as saving time and costs associated with continuous speech therapy sessions conducted via Zoom and Line meetings. Furthermore, the caregivers reported that their children exhibited increased motivation and cooperation during speech therapy sessions using the application.

**Table 4:** Caregivers' satisfaction for speech therapy with Application for Articulation Therapy‐Thai at the end of study.

Satisfaction items	Agreement scores					Average
	Very much: 5, *n* (%)	Much: 4, *n* (%)	Moderate: 3, *n* (%)	A little: 2, *n* (%)	Very little: 1, *n* (%)	
1. Satisfaction with telepractice articulation therapy with AAT‐T	10 (55.56)	7 (38.89)	1 (5.56)	—	—	4.50
2. Telepractice articulation therapy with AAT‐T increases the convenience of getting speech services for speech correction	10 (55.56)	7 (38.89)	1 (5.56)	—	—	4.50
3. Appropriate duration to use telepractice articulation therapy with AAT‐T	12 (66.67)	6 (33.33)	—	—	—	4.67
4. AAT‐T was appropriate for speech correction	14 (77.78)	4 (22.22)	—	—	—	4.78
5. Application Zoom meeting was easy to get telepractice speech therapy	14 (77.78)	4 (22.22)	—	—	—	4.78
6. Stability of picture and sound systems in telepractice articulation therapy with AAT‐T	7 (38.89)	10 (55.56)	1 (5.56)	—	—	4.22
7. Convenience in contact and sending information via the Line application	16 (88.89)	2 (11.11)	—	—	—	4.89
8. Telepractice articulation therapy with AAT‐T reduced the time consumed for speech therapy	16 (88.89)	2 (11.11)	—	—	—	4.89
9. Telepractice articulation therapy with AAT‐T reduced the cost of expense for speech services	15 (83.33)	3 (16.67)	—	—	—	4.83
10. Telepractice articulation therapy with AAT‐T was appropriate for speech correction in the COVID‐19 pandemic	18 (100)	—	—	—	—	5

## 5 Discussion

The use of applications for providing speech therapy has gained significant interest since the lockdowns and quarantines imposed during the COVID‐19 pandemic. The concept of telehealth for rehabilitation services is relatively new, and there are currently no specialty‐specific clinical practice standards or guidelines available to assist rehabilitation practitioners (Almubark et al. 2022). However, advancements in information and communication technologies have created more favorable conditions for delivering remote care across various fields.

A systematic review found that audiovisual materials and interactive videos are highly effective for families participating in interventions. Additionally, the use of educational videos for both children and parents has proven beneficial for facilitating speech correction (Palomares‐Aguilera et al. 2021).

The telepractice application is a safe and reliable tool for addressing articulation disorders. Given that the COVID‐19 pandemic has fundamentally transformed the delivery of healthcare services in the long term, it is essential to explore and implement alternative modes of service delivery (Pamplona and Ysunza 2020).

A previous study demonstrated improvements in articulation speech using both the Turkish Articulation Therapy Application (TARTU) and printed materials (Dural and Ünal–Logacev 2018). In comparing different modalities, one study concluded that telehealth procedures offered significant advantages over non‐telehealth alternatives, achieving an effectiveness rate of 85.5% (Regina molini‐Avejonas et al. 2015).

For the IOC Index, all items of the AAT‐T achieved scores ranging from 0.80 to 1.00, indicating good to excellent content validity. This strong performance can be attributed to the assessment tool's development, which was grounded in sound theoretical foundations related to phonological systems, supported by an extensive literature review, and guided by a rigorous expert process. Furthermore, the exercise items effectively represent Thai universal phonetics, making them suitable for speech correction in Thai children with cleft palate with or without cleft lip (CP ± L).

Regarding face validity, speech‐language pathologists reported satisfaction scores ranging from good to excellent (80%–100%) for functional requirements, achieving an excellent score of 100% for function testing, and an adequate score of 72%–96% for usability testing. Meanwhile, satisfaction scores from children with cleft palate with or without cleft lip (CP ± L) and their caregivers were rated as excellent for functional requirements (92%–100%), function testing (100%), and usability testing (100%). Additionally, the results indicated good ratings for font characteristics (82%) and program responsiveness (88%).

The percentage of agreement between speech‐language pathologists and caregivers was low during the initial use of AAT‐T (first assessment). However, most of these percentages increased significantly after 5 months of practice (second assessment). This improvement in agreement indicates that AAT‐T is a reliable tool for caregivers to report the outcomes of home exercises.

The satisfaction scores revealed that speech‐language pathologists, children with cleft palate with or without cleft lip (CP ± L), and caregivers had significantly high levels of satisfaction with the functionality of AAT‐T. Moreover, all caregivers expressed a strong preference for telemedicine using AAT‐T, citing benefits such as cost savings on living expenses, accommodation, transportation, and compensation, as well as reduced time required to access face‐to‐face speech therapy at the clinic.

AAT‐T offers exercises for each of the 21 initial consonants and 6 final sounds, presented in both pictures and text to elicit target sounds through naming or imitation. There are eight practice levels for each sound, including isolated sounds (via animated videos), as well as four levels of non‐syllable utterances, words, phrases, and both short and long sentences. A speech‐language pathologist, trainer, or caregiver evaluates each practice session by scoring responses as “correct” or “incorrect.” Upon completing each exercise level, the scores are compiled to provide total scores and the percentage of correct and incorrect answers for both naming and imitation modalities, which are interpreted manually.

For implication, AAT‐T shows promise as a valuable tool for speech therapy services. It has the potential to positively impact children's motivation to continue with speech therapy and can serve as a useful aid for speech‐language pathologists (Saeedi et al. 2022).

The application is designed to be attractive, motivational, and easy to use, which may help reduce the number of face‐to‐face therapy sessions required. By utilizing platforms like Zoom (Apple Inc.) and Line (Naver Inc.) for telemedicine, AAT‐T can save time and costs associated with travel to speech clinics. This is especially beneficial for caregivers who may need to take time off work to bring their children to in‐person therapy sessions. As shown in Table 3, the use of AAT‐T significantly reduced the number of articulation errors in this study (Vosburg and Robinson 2022).

In summary, AAT‐T significantly reduced the number of articulation errors. It could positively affect children's motivation to continue speech therapy. The AAT‐T can be used as a speech therapy adjunct tool that can reduce therapist workload and direct treatment sessions to children's homes, enabling them to practice under parental supervision (Saeedi et al. 2022). To establish robust evidence for its efficacy, future research should comprise a large‐scale, prospective randomized controlled trial evaluating the AAT‐T application.

## 6 Conclusion

AAT‐T proved to be a valuable, easy‐to‐use, attractive, motivational, and effective tool for speech therapy in children with cleft palate with or without cleft lip (CP ± L). Furthermore, the use of this application can lead to significant savings in travel expenses, treatment costs, and living expenses. Additionally, AAT‐T has the potential to alleviate the burden on caregivers.

## Author Contributions

Benjamas Prathanee was involved in conceptualization, project administration, design of the study, supervision, assessment, analysis, and drafting the manuscript. Sumita Duangprasert and Sasalaksamon Chanachai were involved in assessing speech and language abilities. Patorn Piromchai was involved in proposal design, analysis, and revised the final manuscript. All authors approved the final manuscript.

## Conflicts of Interest

The authors declare no conflicts of interest.

## Data Availability

The data that support the findings of this study are available on request from the corresponding author. The data are not publicly available due to privacy or ethical restrictions.
